# Nonlinear association of HbA1c/HDL-C ratio with heart failure in adults: a large cross-sectional study

**DOI:** 10.3389/fcvm.2026.1844807

**Published:** 2026-06-03

**Authors:** Miao Li, Mei Hu, Xujue Ma, Zhuoga Ciren, Dengzhu Cideng, Guangsu Qu, Yangcuo Silang

**Affiliations:** 1 Department of Cardiology, The Second Affiliated Hospital of Chongqing Medical University, Chongqing, China; 2 Department of Cardiology, Changdu People's Hospital of Xizang, Xizang, China; 3 Department of Critical Care Medicine, The First Affiliated Hospital of Chongqing Medical and Pharmaceutical College, Chongqing, China

**Keywords:** hbA1c/HDL-C ratio, heart failure, inpatients, NHANES, nonlinear association, restricted cubic spline

## Abstract

**Background:**

Heart failure (HF) remains a leading cause of morbidity and mortality worldwide. The HbA1c/HDL-C ratio, a novel composite biomarker reflecting glycemic control and lipid metabolism, may be associated with HF risk, but its specific relationship remains underexplored. This study aimed to investigate the association between the HbA1c/HDL-C ratio and HF.

**Methods:**

We utilized samples from two sources: 1,360 inpatients from a cardiology department in 2020 and 101,316 participants from the National Health and Nutrition Examination Survey (*N*HANES) 1999–2018. Multivariate logistic regression, subgroup analyses, restricted cubic spline (RCS) regression, and receiver-operating characteristic (ROC) curve analysis were employed to examine the relationship.

**Results:**

In an analysis of the NHANES sample including 48,922 participants, 1,598 individuals were identified as having HF, corresponding to a prevalence of 3.27%. In a separate clinical sample of 584 participants, 132 had HF, yielding an overall prevalence of 22.60%. After full adjustment, the HbA1c/HDL-C ratio demonstrated a consistent positive association with HF risk in both the NHANES sample (OR =1.07, 95% CI: 1.01–1.14; *P* = 0.033) and the clinical sample (OR = 1.15, 95% CI: 1.01–1.30; *P* = 0.029). Furthermore, a nonlinear relationship between the HbA1c/HDL-C ratio and HF risk was observed in both populations, with an inflection point identified at 6.21. Below this threshold (HbA1c/HDL-C < 6.21), the adjusted OR was 1.22 (95% CI: 1.09–1.37; *P* = 0.001) in the NHANES sample and 1.65 (95% CI: 1.20–2.28; *P* = 0.002) in the clinical sample. Above the inflection point (HbA1c/HDL-C ≥ 6.21), the association attenuated and became statistically nonsignificant. The ROC curve analysis also confirmed that the HbA1c/HDL-C ratio combination had more robust predictive power for heart failure than HbA1c and HDL-C alone (*P* < 0.05).

**Conclusion:**

This study found a nonlinear positive association between the HbA1c/HDL-C ratio and the risk of HF, identifying key threshold values.

## Introduction

Heart failure (HF) remains a major global health burden (1), currently affecting an estimated 64 million individuals worldwide, with prevalence projected to rise further as populations age and risk factors exposure increases (2, 3). Despite substantial advances in pharmacologic and device-base therapies, HF continues to be associated with high morbidity and mortality, highlighting the persistent need for improved risk stratification, early identification of at-risk individuals, and preventive interventions that target upstream pathophysiology (2, 4). The clinical phenotype of HF reflects a convergence of diverse etiologies and remodeling processes, wherein metabolic disturbances play a central and potentially unifying role across HF with reduced ejection fraction (HFrEF) and HF with preserved ejection fraction (HFpEF) (2).

Glycated hemoglobin (HbA1c) remains the gold standard biomarker for assessing long-term glycemic control, reflecting average blood glucose over roughly the preceding 2–3 months, and its elevation is consistently linked with higher risk of adverse cardiovascular outcomes, including HF and the development of diabetic cardiomyopathy (5–7). Among individuals with diabetes or prediabetes, each 1% absolute increase in HbA1c has been associated with a substantially higher risk of HF (8). Evidence from multiple large cohorts and meta-analysis further support a positive, non-linear (often J- or U -shaped) relationship between HbA1c and HF risk, suggesting that both inadequate glycemic control and, in certain contexts, overly intensive glucose lowering may fail to confer cardiovascular benefit and may even be detrimental (9). In patients with established HF, the relationship between HbA1c and clinical outcomes appears more complex and may vary according to diabetes status and HF phenotype (HFrEF vs. HFpEF) (10). Some analyses have reported improved short- to mid-term among patients with diabetes and HF who maintain modest HbA1c levels, whereas others have observed a U-shaped association, with the best outcomes at intermediate HbA1c ranges (approximately 7%) (11).

High-density lipoprotein cholesterol (HDL-C) has long been recognized for cardioprotective properties, including its central role in reverse cholesterol transport, as well as its anti-inflammatory and antioxidant effects, which collectively contribute to the attenuation of atherogenesis and vascular injury (12). In HF and broader cardiovascular cohorts, lower HDL-C levels are consistently associated with greater risk of adverse outcomes, including HF hospitalization and mortality, suggesting that HDL-C serves as an independent marker of cardiovascular risk beyond traditional factors (13, 14). Although the protective role of HDL-C is well-supported,accumulating evidence indicates that HDL quality and functionality—beyond merely circulating HDL-C—may influence risk. In certain HF populations, the strength of the association between HDL-C and clinical outcomes may be attenuated by concurrent metabolic disturbances or statin/antidiabetic therapies (15).

Recently, composite biomarkers have attracted increasing attention for their capacity to provide a more holistic view of metabolic health than single parameters (16, 17). The HbA1c/HDL-C ratio has been demonstrated to integrate information regarding both glucose control and lipid metabolism.An elevated ratio may reflect poor glycemic regulation, reduced levels of protective lipoproteins, or the coexistence of both abnormalities, thereby representing a compounded metabolic derangement that may be particularly deleterious to myocardial structure and function.However, the specific nature of the association between the HbA1c/HDL-C ratio and HF remains to be fully elucidated.

The present study hypothesised that the HbA1c/HDL-C ratio is independently associated with HF in adults. Utilising two distinct samples (a single-center inpatient sample and a large, nationally representative NHANES sample), the objective was to validate this association and explore its specific trends and potential thresholds.

## Methods

### Study population

The present study utilized data from two distinct sources. The primary dataset was obtained from the NHANES, a nationally representative surveillance program conducted by the National Center for Health Statistics (NCHS). The NCHS employs a multistage stratified probability sampling design to ensure the representation of the U.S. civilian non-institutionalized population (18, 19). The study protocol was approved by the NCHS institutional review board, and all participants provided written informed consent (20). Furthermore, clinical data were retrospectively collected from the electronic medical records of patients admitted to the Department of Cardiology at Xizang Changdu City People's Hospital in 2020. This component of the study was reviewed and approved by the Institutional Ethics Committee (Approval No. 2025008).

**Figure 1:**
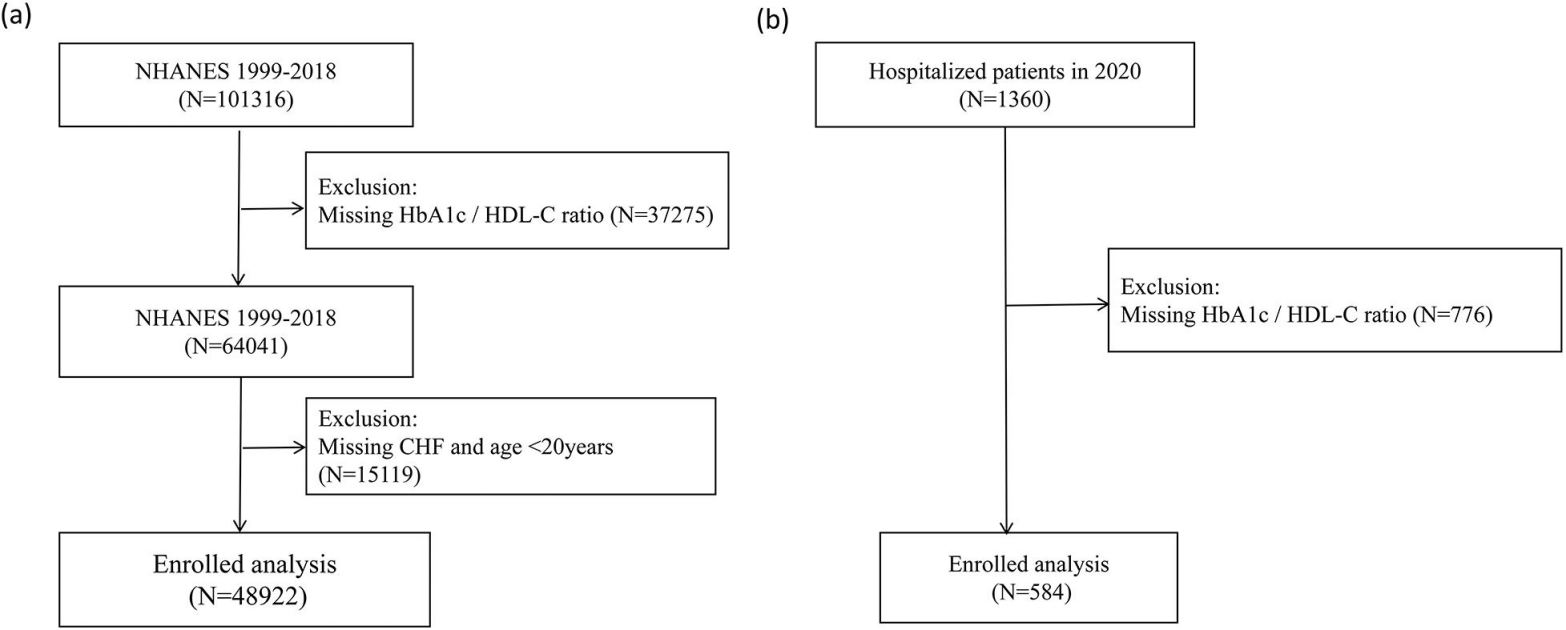
Flow chart of study sample screening in NAHNES **(a)** and clinical data **(b)**.

As illustrated in Figure 1A, the data were derived from NHANES data collected between 1999 and 2018, comprising an initial sample of 101,316 participants. Exclusions were applied sequentially in the following order: first, 37,275 participants without HbA1c or HDL-C data were excluded; next, 15,119 individuals lacking HF data and younger than 20 years were excluded. Subsequent to the application of these exclusions, the analytic sample comprised 48,922 participants.An additional set of data was obtained from inpatients in a hospital cardiology department (Figure 1B). The initial sample comprised 1,360 patients in 2020. Exclusions due to missing HbA1c or HDL-C ratio totaled 776, yielding an analytic sample of 584 patients.

### Definitions of exposure and outcome variables

The HbA1c–to–HDL-C ratio was defined as a novel composite biomarker representing the integration of glycemic regulation and lipid homeostasis, calculated as HbA1c (%) divided by HDL-C(mmol/L) (21).The definition of HF in this study was based on a combination of self-reported data and clinical assessments. In the NHANES screening, HF was defined as a positive response to the question, “Has a doctor or health professional ever told you that you have CHF?” Participants were eligible for inclusion if they met two criteria: an age of 20 years of older and the availability of complete data for analysis. To enhance diagnostic accuracy, NHANES incorporates self-reported information, clinical assessments, and a review of medical records (22).

### Potential covariates

Baseline covariates collected from NHANES participants encompassed demographic and socioeconomic characteristics, including age (years), sex (male or female), ethnicity (non-Hispanic White, non-Hispanic Black, Mexican American, or other race), marital status (married or living with a partner; widowed, divorced, or separated; never married), poverty-to-income ratio(PIR), and education level (less than high school; high school graduate; above high school). Behavioral factors examined in this study comprised smoking status and drinking status, each categorized as: never, now, and former. Participants’ alcohol consumption was assessed using a standardized questionnaire and categorized by self-reported intake into three groups: (1) Never drinkers (<12 lifetime drinks), (2) Former drinkers (≥12 lifetime drinks, but none in the past 12 months), and (3) Current drinkers (≥12 drinks in the past 12 months) (23, 24). Smoking behavior was categorized into three groups: current smokers (participants who currently smoke one or more cigarettes daily), former smokers (those who had smoked more than 100 cigarettes in their lifetime but had quit), and never smokers (those who had smoked fewer than 100 cigarettes in their lifetime) (25). Documented diseases included hypertension, diabetes mellitus (DM), coronary heart disease (CHD), and stroke, with most information on hypertension, CHD, and stroke derived from self-report. Baseline laboratory measurements included total cholesterol (TC, mmol/L) and neutrophil count (×10^^9^/L).

The clinical data included in the study was retrieved from electronic medical records and comprised the following covariates: age (in years); sex (male or female); education level (less than high school, high school, or above high school); marital status (never married, married, or widowed/divorced); and smoking and alcohol consumption status (never, current, or former). The diagnoses recorded included hypertension, DM, CHD, and stroke. The anthropometric and laboratory variables encompassed the following metrics: body mass index (BMI, kg/m^2^); fasting low-density lipoprotein cholesterol (LDL-C, mmol/L); and uric acid (µmol/L).

### Statistical analysis

Descriptive analyses were conducted to summarize the baseline characteristics of all participants. Statistical analyses were performed using R software (version 4.1.3) and Empower Stats (version 2.0), employing two-sided tests and a significance level of *P* < 0.05. The study adhered to the Strengthening the Reporting of Observational Studies in Epidemiology (STROBE) guidelines.Continuous variables were presented as means ± standard deviation (SD) or medians with interquartile range (IQR), as appropriate, and were compared using t-tests. Categorical variables were reported as percentages and were analyzed with the chi-square test. Subsequently, multivariate logistic regression models were employed to examine the association between the HbA1c/HDL-C ratio and HF, with three models in NHANES data. The following three models were specified for the clinical data: Model 1 (unadjusted), Model 2 (adjusted for age and gender), and Model 3 (fully adjusted for age, gender, marital status, education level, smoking behavior, alcohol consumption, hypertension, DM, BMI, LDL-C, stroke, uric acid, and CHD). To further explore potential nonlinear relationships between the HbA1c/HDL-C ratio and HF, a restricted cubic spline (RCS) model was fitted within the multivariable logistic regression framework, adjusting for all covariates included in Model 3. Furthermore, the study evaluated the dose-response relationships between the HbA1c/HDL-C ratio and outcome variables using smooth curve fitting within a standard linear framework, complemented by threshold analysis via a two-piecewise linear model. The presence of a threshold was assessed using a likelihood ratio test, and a two-step recursive method identified the inflection point. A linear association was indicated by *P* < 0.05 in the standard linear model, and the existence of a threshold was confirmed when the *P*-value from the likelihood ratio test was also < 0.05. Finally, a subgroup analysis was conducted to examine potential heterogeneity across multiple demographic and clinical characteristics. Interaction tests were used to assess the statistical significance of interactions, and *P*-values were reported for each analysis. The predictive power of the indices for HF risks was evaluating using receiver operating characteristic (ROC) curve analysis. For joint assessment, both the HbA1c and HDL-C were treated as continuous variables and included simultaneously in the predictive model. The area under the curve (AUC) was calculated to compare the predictive capacity of the HbA1c, HDL-C and the combined HbA1c/HDL-C ratio assessment.

To ensure the robustness of our findings, we performed several sensitivity analyses. First, to address missing covariate data and minimize potential bias while maximizing statistical power, we employed multiple imputation using the Markov-Chain Monte Carlo method within the SAS multiple imputation procedure. Additionally, to further clarify the roles of insulin resistance and hyperinsulinemia in the elevated risk of HF, we evaluated the association between the HbA1c/HDL-C ratio and HF within strata defined by diabetes status. This involved conducting both stratification analysis and interaction tests within the regression model.

## Results

### Baseline characteristics

Table 1 demonstrates the baseline characteristics of 48,922 participants from NHANES 1999–2018 grouped by HF. A total of 1,598 participants had HF, with a prevalence of 3.27%. The mean age of all participants was 49.60 ± 18.16 years, with 48.21% males and 51.79% females.Compared with individuals without HF, those with HF were significantly older and exhibited a higher HbA1c/HDL-C ratio and neutrophil count, accompanied by lower total cholesterol levels (all *P* < 0.001).The HF group was more frequently male and more commonly identified as non-Hispanic White or Black, and participants were more likely to be widowed, divorced, or separated. In addition, individuals with HF tended to have lower income and educational attainment. We conducted further analyses comparing baseline characteristics between included and excluded participants. The results showed that despite some differences, the overall distributions of key demographic and clinical variables were broadly comparable (Supplementary Table S1).

**Table 1:** Baseline characteristics of NHANES participants.

Characteristic	Total(*n* = 48922)	Non-HF(*n* = 47324)	HF(*n* = 1598)	*P*-value
HbA1c/HDL-C Ratio	4.58 ± 1.81	4.55 ± 1.78	5.55 ± 2.31	
Age (years)	49.60 ± 18.16	48.99 ± 18.00	67.71 ± 12.67	<0.001
TC, mg/dL	203.66 ± 43.32	203.91 ± 43.20	195.88 ± 46.32	<0.001
Neutrophil, 10^9^/L	4.32 ± 1.79	4.31 ± 1.79	4.68 ± 1.83	<0.001
Gender,n(%)				<0.001
Male	23584 (48.21)	22685 (47.94)	899 (56.26)	
Female	25338 (51.79)	24639 (52.06)	699 (43.74)	
Ethnicity,n(%)				<0.001
Non-Hispanic White	21848 (44.66)	20975 (44.32)	873 (54.63)	
Non-Hispanic Black	9866 (20.17)	9498 (20.07)	368 (23.03)	
Mexican American	8682 (17.75)	8507 (17.98)	175 (10.95)	
Other Race	8526 (17.43)	8344 (17.63)	182 (11.39)	
Marital status,n(%)				<0.001
Married/Living with Partner	29377 (60.05)	28575 (60.38)	802 (50.19)	
Widowed/Divorced/Separated	10698 (21.87)	10036 (21.21)	662 (41.43)	
Never married	8374 (17.12)	8255 (17.44)	119 (7.45)	
Missing	473 (0.97)	458 (0.97)	15 (0.94)	
PIR,n(%)				<0.001
Poor	9089 (18.58)	8729 (18.45)	360 (22.53)	
Nearly poor	11961 (24.45)	11419 (24.13)	542 (33.92)	
Middle income	12008 (24.55)	11631 (24.58)	377 (23.59)	
High income	11622 (23.76)	11442 (24.18)	180 (11.26)	
Missing	4242 (8.67)	4103 (8.67)	139 (8.70)	
Education level,n(%)				<0.001
Below high school	5897 (12.05)	5581 (11.79)	316 (19.77)	
High school	18554 (37.93)	17849 (37.72)	705 (44.12)	
Above high school	24407 (49.89)	23833 (50.36)	574 (35.92)	
Missing	64 (0.13)	61 (0.13)	3 (0.19)	
Drinking behavior,n(%)				<0.001
Never	6437 (13.16)	6212 (13.13)	225 (14.08)	
Former	7807 (15.96)	7267 (15.36)	540 (33.79)	
Now	34678 (70.88)	33845 (71.52)	833 (52.13)	
Missing	4874 (9.96)	4698 (9.9)	176 (11.0)	
Smoking behavior,n(%)				<0.001
Never	26555 (54.28)	25935 (54.80)	620 (38.80)	
Former	12151 (24.84)	11472 (24.24)	679 (42.49)	
Now	10174 (20.80)	9876 (20.87)	298 (18.65)	
Missing	42 (0.09)	41 (0.09)	1 (0.06)	
DM,n(%)				<0.001
Yes	8459 (17.78)	7696 (16.74)	763 (47.81)	
Borderline	3452 (7.26)	3336 (7.26)	116 (7.27)	
Hypertension,n(%)	20416 (41.74)	19102 (40.37)	1314 (82.23)	<0.001
CHD,n(%)	2015 (4.13)	1372 (2.91)	643 (41.56)	<0.001
Stroke,n(%)	1884 (3.85)	1546 (3.27)	338 (21.20)	<0.001

For continuous variables: survey-weighted mean (95% CI), *P*-value was by survey-weighted linear regression.

For categorical variables: survey-weighted percentage (95% CI), *P*-value was by survey-weighted Chi-square test.

Q, quartile; TC, total cholesterol (mg/dL); DM, diabetes mellitus; CHD, coronary heart disease; HF, heart failure.

As shown in Table 2, the study included 584 hospitalized patients in 2020, of whom 132 (22.6%) were diagnosed with HF. The mean age of the participants was 62.27 ± 14.91 years, and the cohort consisted of 296 males (50.68%) and 288 females (49.32%).HF patients were older and had higher levels of LDL-C and a higher HbA1c/HDL-C ratio, together with a greater prevalence of hypertension, prior HF, and prior stroke. Conversely, uric acid levels were lower in the HF group. Education level and marital status also diverged significantly (*P* < 0.05). No significant differences were observed in BMI, gender, or smoking and drinking behaviors between the groups.

**Table 2:** Baseline characteristics of clinical participants.

Characteristic	Total(*n* = 584)	Non-HF(*n* = 452)	HF(*n* = 132)	*P*-value
Age(years)	62.27 ± 14.91	60.16 ± 14.86	69.48 ± 12.68	<0.001
BMI(kg/m^2^)	24.78 ± 3.76	24.78 ± 3.73	24.77 ± 3.91	0.970
LDL-C(mg/dL)	356.42 ± 111.04	346.89 ± 102.21	388.89 ± 132.30	<0.001
Uric(umol/L)	2.43 ± 0.92	2.49 ± 0.90	2.22 ± 0.98	0.003
HbA1c/HDL-C Ratio	6.07 ± 2.85	5.90 ± 2.83	6.66 ± 2.85	0.007
Gender,n(%)				0.828
Male	296 (50.68)	228 (50.44)	68 (51.52)	
Female	288 (49.32)	224 (49.56)	64 (48.48)	
Education level,n(%)				<0.001
Less than high school	32 (5.48)	7 (5.30)	25 (5.53)	
High school	501 (85.79)	107 (81.06)	394 (87.17)	
Above high school	51 (8.73)	18 (13.64)	33 (7.30)	
Marital status,n(%)				0.032
Never married	321 (54.97)	94 (71.21)	227 (50.22)	
Married	138 (23.63)	22 (16.67)	116 (25.66)	
Widowed/Divorced	125 (21.40)	16 (12.12)	109 (24.12)	
Smoking behavior,n(%)				0.064
Never	434 (74.32)	327 (72.35)	107 (81.06)	
Now	112 (19.18)	96 (21.24)	16 (12.12)	
Fomer	38 (6.51)	29 (6.42)	9 (6.82)	
Drinking behavior,n(%)				0.214
Never	470 (80.48)	361 (79.87)	109 (82.58)	
Now	97 (16.61)	80 (17.70)	17 (12.88)	
Fomer	17 (2.91)	11 (2.43)	6 (4.55)	
Hypertension,n(%)	386 (66.10)	284 (62.83)	102 (77.27)	0.002
DM,n(%)	200 (34.25)	150 (33.19)	50 (37.88)	0.318
HF(%)	246 (42.12)	168 (37.17)	78 (59.09)	<0.001
Stroke(%)	44 (7.53)	27 (5.97)	17 (12.88)	0.008

For continuous variables: survey-weighted mean (95% CI), *P*-value was by survey-weighted linear regression.

For categorical variables: survey-weighted percentage (95% CI), *P*-value was by survey-weighted Chi-square test.

HDL-C, low-density lipoprotein cholesterol; DM, diabetes mellitus; CHD, coronary heart disease; HF, heart failure.

### Association between HbA1c/HDL-C ratio and HF

As illustrated in Table 3, the HbA1c/HDL-C ratio was positively associated with the risk of HF in the NHANES population.In continuous analysis, each unit increment in the HbA1c/HDL-C ratio was associated with higher odds of HF across all three models: Model 1 (OR =1.22, 95% CI 1.20–1.25; *P* < 0.001), Model 2 (OR =  1.21, 95% CI 1.19–1.24; *P* < 0.001), and Model 3 (OR = 1.07, 95% CI 1.01–1.14; *P* = 0.033). Quartile-based categorical analysis demonstrated a graded dose-response relationship. Participants in the highest quartile (Q4) had significantly greater odds of HF than those in the lowest quartile (Q1) in all models: Model 1 (OR =  3.53, 95% CI 3.02–4.11; *P* < 0.001), Model 2 (OR =  3.13, 95% CI 2.66–3.67; *P* < 0.001), and Model 3 (OR =1.66, 95% CI 1.13–2.45; *P* = 0.001).The significant *P* for trend (<0.001) across all models further confirmed this graded association, although the strength of association was attenuated in the fully adjusted Model 3, particularly for the middle quartiles. HDL-C was significantly inversely associated with HF across all models (*P* < 0.01), with higher quartiles of HDL-C demonstrating significantly lower odds of HF (*P* for trend < 0.001). For continuous HbA1c, a significant positive association with HF was observed in all models (*P* < 0.01). While a strong dose-response was evident in Model 1, in Models 2 and 3, primarily the highest quartile (Q4) of HbA1c showed a significant association with HF (*P* < 0.05), with overall significant *P* for trend across all models (*P* < 0.001).

**Table 3:** Logistic regression between **HbA1c/HDL-C ratio and HF** (NHANES).

Variables	Model1		Model2		Model3	
	OR(95%CI)	*P* value	OR(95%CI)	*P* value	OR(95%CI)	*P* value
**HbA1c/HDL-C ratio**						
Continuous	1.22 (1.20, 1.25)	<0.001	1.21 (1.19, 1.24)	<0.001	1.07 (1.01, 1.14)	0.033
Quartiles						
Q1	Reference		Reference		Reference	
Q2	1.25 (1.04, 1.50)	0.016	1.25 (1.04, 1.50)	0.018	1.15 (0.77, 1.70)	0.495
Q3	1.78 (1.50, 2.10)	<0.001	1.71 (1.44, 2.04)	<0.001	1.39 (0.95, 2.04)	0.087
Q4	3.53 (3.02, 4.11)	<0.001	3.13 (2.66, 3.67)	<0.001	1.66 (1.13, 2.45)	0.001
*P* for trend		0.001		0.001		0.001
**HDL-C**						
Continuous	0.47 (0.41, 0.54)	<0.001	0.39 (0.34, 0.45)	<0.001	0.60 (0.43, 0.84)	0.003
Quartiles						
Q1	Reference		Reference		Reference	
Q2	0.67 (0.59, 0.77)	<0.001	0.63 (0.55, 0.72)	<0.001	0.64 (0.48, 0.86)	0.003
Q3	0.53 (0.47, 0.61)	<0.001	0.49 (0.42, 0.56)	<0.001	0.66 (0.48, 0.90)	0.009
Q4	0.45 (0.39, 0.52)	<0.001	0.37 (0.32, 0.43)	<0.001	0.56 (0.40, 0.80)	0.001
*P* for trend		0.001		0.001		0.001
**HbA1c**						
Continuous	1.34 (1.30, 1.38)	<0.001	1.25 (1.21, 1.30)	<0.001	1.04 (1.02, 1.10)	0.002
Quartiles						
Q1	Reference		Reference		Reference	
Q2	1.40 (1.12, 1.74)	0.002	0.89 (0.71, 1.11)	0.311	0.86 (0.58, 1.29)	0.465
Q3	2.33 (1.90, 2.87)	<0.001	1.07 (0.86, 1.32)	0.556	1.10 (0.74, 1.64)	0.648
Q4	5.63 (4.69, 6.77)	<0.001	1.91 (1.58, 2.31)	<0.001	1.47 (1.01, 2.15)	0.040
*P* for trend		0.001		0.001		0.001

Q, Quartile; TC, Total Cholesterol (mg/dL); DM, Diabetes Mellitus; CHD, Coronary Heart Disease; OR, odds ratio, CI, confidence interval.

Model 1: Non-adjusted model.

Model 2: Adjusted for age, gender.

Model 3: Adjusted for age, gender, ethnicity, marital status, poverty income ratio, education level, smoking behavior, drinking behavior, hypertension, DM, Stroke, neutrophils, TC, CHD.

Table 4 presents the examining the association between HbA1c/HDL-C ratio and clinical HF across three progressive models. When analyzed as a continuous variable, each unit increase in HbA1c/HDL-C ratio was significantly associated with higher odds of HF in all three models (Model 1: OR = 1.09, 95%CI 1.02–1.16, *P* = 0.009; Model 2: OR = 1.12, 95%CI 1.05–1.20, *P* = 0.001; Model 3: OR = 1.15, 95%CI 1.01–1.30, *P* = 0.029). Similarly, tertile analysis demonstrated a significant positive trend across increasing tertiles (*P* for trend <0.001 for all models), with the highest tertile (T3) consistently showing significantly elevated odds of HF compared to the reference group (T1) across all three models (Model 1: OR = 2.48, 95%CI 1.50–4.89, *P* = 0.001; Model 2: OR = 2.87, 95%CI 1.68–4.93, *P* = 0.001; Model 3: OR = 2.39, 95%CI 1.15–4.96, *P* = 0.019), while the second tertile (T2) showed marginal or non-significant associations depending on the model adjustment. Conversely, HDL-C demonstrated a significant inverse association with HF. Regarding HbA1c, no significant association with HF was found when treated as a continuous variable. However, the highest tertile (T3) of HbA1c was significantly associated with increased odds of HF in Model 1 (*P* = 0.034) and Model 3 (*P* = 0.028), with a significant trend observed in these two models (*P* < 0.033).

**Table 4:** Logistic regression between **HbA1c/HDL-C ratio and HF** (clinical).

Variables	Model1		Model2		Model3	
	OR(95%CI)	*P* value	OR(95%CI)	*P* value	OR(95%CI)	*P* value
**HbA1c/HDL-C ratio**						
Continuous	1.09 (1.02, 1.16)	0.009	1.12 (1.05, 1.20)	0.001	1.15 (1.01, 1.30)	0.029
Tertile						
T1	Reference		Reference		Reference	
T2	1.67 (1.00,2.81)	0.049	1.65 (0.96,2.86)	0.069	1.53 (0.79,2.95)	0.199
T3	2.48 (1.50,4.89)	0.001	2.87 (1.68,4.93)	0.001	2.39 (1.15,4.96)	0.019
*P* for trend		0.001		0.001		0.001
**HDL-C**						
Continuous	0.23 (0.12, 0.46)	<0.001	0.20 (0.10, 0.42)	<0.001	0.21 (0.08, 0.53)	0.001
Tertile						
T1	Reference		Reference		Reference	
T2	0.53 (0.34, 0.85)	0.007	0.55 (0.34, 0.90)	0.017	0.64 (0.35, 1.17)	0.146
T3	0.38 (0.23, 0.62)	0.001	0.37 (0.22, 0.62)	0.001	0.39 (0.20, 0.77)	0.006
*P* for trend		<0.001		<0.001		0.006
**HbA1c**						
Continuous	0.99 (0.91, 1.08)	0.851	1.03 (0.94, 1.14)	0.530	1.04 (0.90, 1.21)	0.604
Tertile						
T1	Reference		Reference		Reference	
T2	1.01 (0.60, 1.69)	0.978	0.76 (0.44, 1.31)	0.328	0.89 (0.45, 1.76)	0.747
T3	1.70 (1.04, 2.79)	0.034	1.47 (0.88, 2.47)	0.141	2.07 (1.10, 5.54)	0.028
*P* for trend		0.022		0.073		0.033

T, Tertile; DM, Diabetes Mellitus; CHD, Coronary Heart Disease; HDL-C, low-density lipoprotein cholesterol; OR, odds ratio, CI, confidence interval.

Model 1: Non-adjusted model.

Model 2: Adjusted for age, gender.

Model 3: Adjusted for age, gender, marital status, education level,smoking behavior, drinking behavior, hypertension, DM, BMI,LDL-C,Stroke, uric, CHD.

### Nonlinear relationship between HbA1c/HDL-C ratio and HF

**Figure 2:**
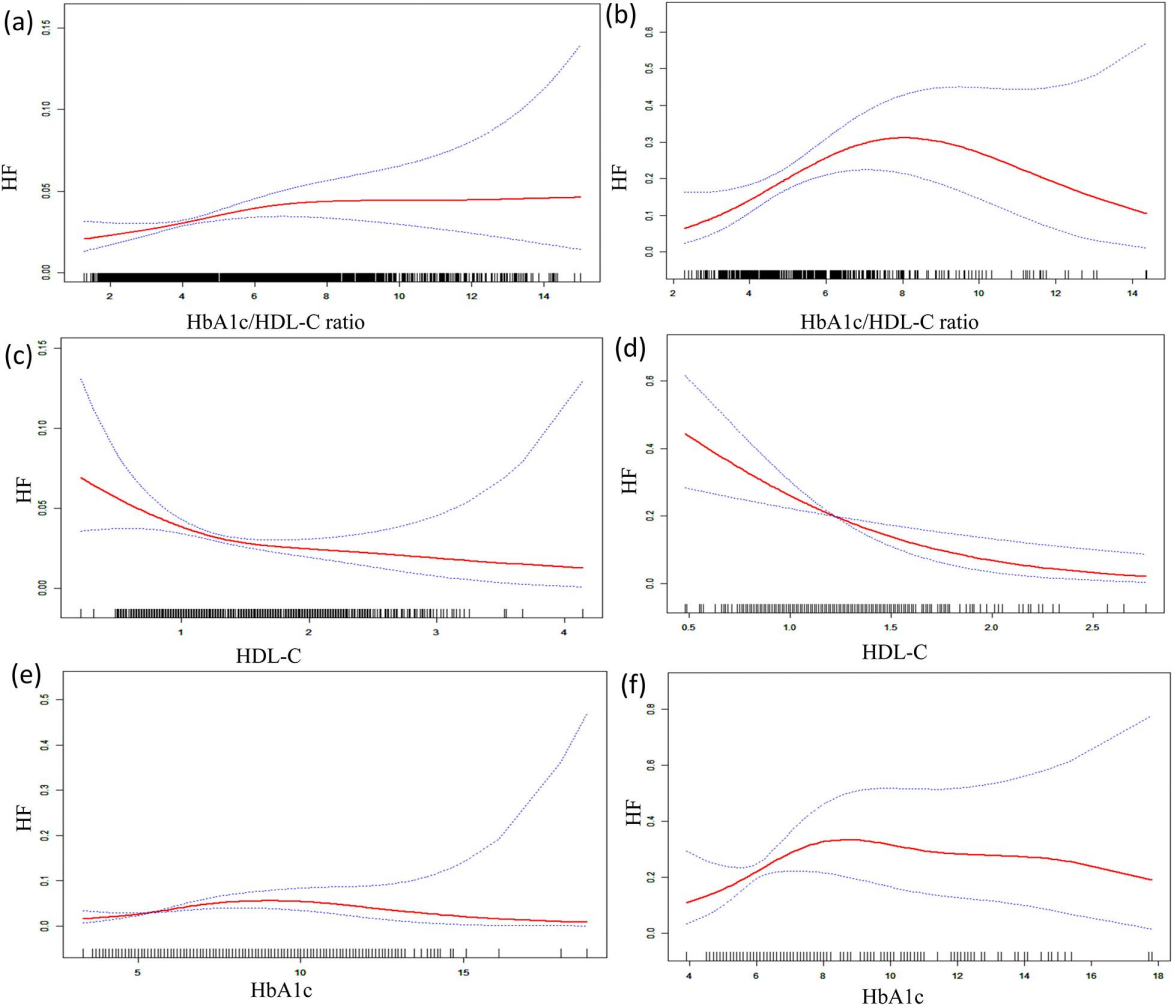
Restricted cubic spline (RCS) curves for the associations of HbA1c/HDL-C ratio, HbA1c, and HDL-C with HF. Panel **(a) (c) (e)** shows RCS curves from the NHANES data, and panel **(b) (d) (f)** shows RCS curves from the clinic data. The red and blue dotted lines represent the estimated values and their corresponding 95% CIs. Adjusted for the variables listed in model 3.

As depicted in Figure 2, complex and frequently non-linear associations were observed between the HbA1c/HDL-C ratio, HDL-C, and HbA1c with the risk of heart failure (HF). Specifically, an elevated HbA1c/HDL-C ratio demonstrated an association with increased HF risk (Figures 2A,B). Conversely, HDL-C levels exhibited an inverse relationship with HF risk, indicating that higher HDL-C levels correlated with a reduced probability of HF (Figures 2C,D). Furthermore, HbA1c levels presented a non-linear association with HF risk, wherein a significant elevation in HF risk was observed at higher HbA1c concentrations (Figures 2E,F). The HbA1c/HDL-C ratio demonstrated a nonlinear association with HF risk, with an inflection point at 6.21. In the NHANES data, the adjusted OR was 1.22 (95% CI: 1.09,1.37; *P* = 0.001), and in the clinical data, it was 1.65 (95% CI: 1.20,2.28; *P* = 0.002). Across both data sets, the HF risk exhibited an increase as the HbA1c/HDL-C ratio approached 6.21, with a subsequent plateau or attenuation beyond the inflection point (Table 5).

**Table 5:** Threshold effect analysis. Adjusted for the variables listed in model 3.

Models	NHANES data	Clinical data
	OR(95% CI) *P* value	OR(95% CI) *P* value
Fitting by the standard linear model	1.07 (1.01, 1.14) 0.033	1.14 (0.98, 1.31) 0.083
Fitting by the two-piecewise linear model		
Inflection point	6.21	6.21
<Inflection point	1.22 (1.09, 1.37) 0.001	1.65 (1.20, 2.28) 0.002
>Inflection point	0.96 (0.86, 1.07) 0.477	0.90 (0.71, 1.14) 0.389
*P* for Log-likelihood ratio	0.007	0.007

### Subgroup analysis and sensitivity analysis

**Figure 3:**
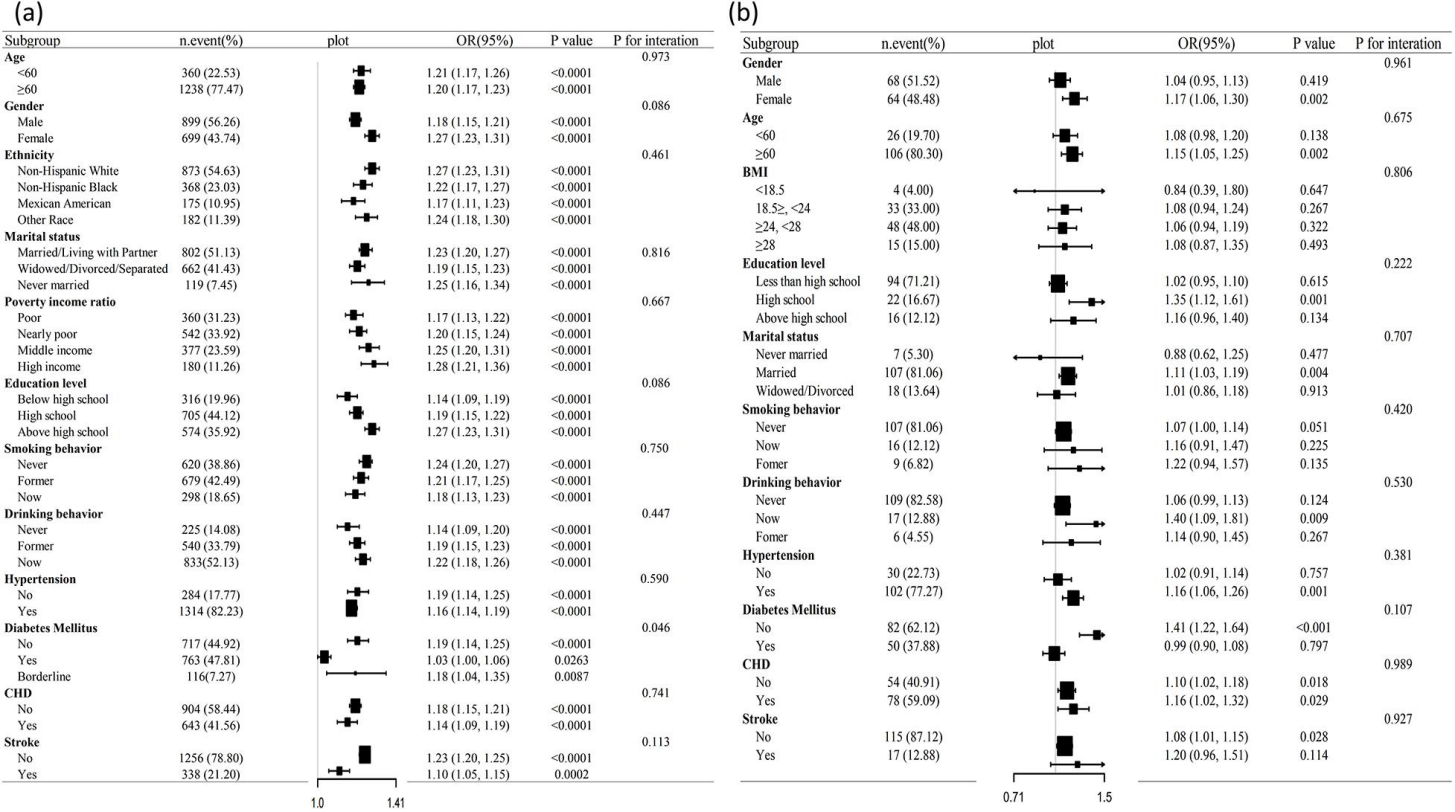
Subgroup analysis of the association between the HbA1c/HDL-C ratio and HF. Adjusted for the variables listed in model 3 except for the variable used for stratification. (**a**) NHANES data and (**b**) Clinical data.

In order to examine whether the association remained consistent across various demographic and health-related variables, subgroup analyses of NHANES data were performed (Figure 3A). The results indicated that statistically significant positive associations were observed in nearly all subgroups (all *P* < 0.0001), and the association remained consistent across variables such as age, sex, ethnicity, marital status, history of hypertension, DM, CHD, and stroke. No significant interactions of these stratified variables were observed (all *P* for interaction >0.05). However, a notable interaction was identified for DM status (*P* for interaction=0.046), with a more pronounced association observed among participants with diabetes.

Clinical data analysis revealed that subgroup analyses were performed stratified by gender, age, BMI, education level, marital status, smoking behavior, drinking behavior, hypertension, DM, CHD, and stroke (Figure 3B). The results indicated a consistent positive association between the HbA1c/HDL-C ratio and HF across most subgroups. However, no significant interactions were observed across any of the stratified subgroups (all *P* for interaction > 0.05).

In a sensitivity analysis, we further performed subgroup analyses stratified by diabetes status. It was found that the significant positive correlation between the HbA1c/HDL-C ratio and the risk of heart failure persisted in both diabetic and non-diabetic individuals (Supplementary Table S2). Concurrently, a reanalysis of the association between the HbA1c/HDL-C ratio and heart failure using imputation data qualitatively supported the primary findings (Supplementary Table S3).

### ROC curve analysis of HbA1c, HDL-C, and HbA1c/HDL-C ratio

Further ROC analysis and AUC comparison revealed that the HbA1c/HDL-C ratio exhibited enhanced predictive capability for HF risk compared to either individual HbA1c or HDL-C. Specifically, our results indicated that although both individual markers were associated with HF risk, the HbA1c/HDL-C ratio demonstrated a more pronounced and consistent association (Supplementary Figure S1).

## Discussion

This cross-sectional study aims to comprehensively investigate the association between the HbA1c/HDL-C ratio and HF by analyzing two independent study populations. Our primary findings reveal a significant, robust, and distinctly nonlinear positive association between the HbA1c/HDL-C ratio and HF risk. A threshold value of 6.21 was identified, beyond which the risk of HF either plateaus or increases at a significantly slower rate, suggesting a potential saturation effect in the metabolic pathways contributing to HF pathogenesis.

Among 65,950 patients with HF, HbA1c variability (assessed by average successive variability or standard deviation) was significantly associated with increased risks of HF rehospitalization and all-cause mortality (26).In a large cohort involving 271,174 diabetic patients and 1,355,870 matched controls found that while keeping five key risk factors-glycated hemoglobin, low-density lipoprotein cholesterol, albuminuria, smoking, and blood pressure-within recommended target ranges was associated with normalization of the risk of death, acute myocardial infarction, and stroke, the risk of hospitalization for HF remained significantly higher (27).

Several studies have revealed the complex relationship between HDL-C levels, HF and mortality.In a retrospective cohort study of 810,848 Korean adults, HDL-C levels ≤40 mg/dL,as compared with levels of 40–60 mg/dL,were associated with higher risks of all-cause mortality, cardiovascular death, ischemic heart disease, ischemic stroke, and HF (28). In a study of 15.8 million Korean adults without a history of CVD or cancer, baseline HDL-C showed a U-shaped association with CVD mortality during a median follow-up of 8.8 years. When HDL-C levels were below 60 mg/dL, each increase of 39 mg/dL in HDL-C was linked to a 42% lower risk of CVD death. However, within the range of 60–150 mg/dL, higher HDL-C levels were associated with increased risk, particularly for HF mortality (HR = 1.20) (29).In a retrospective analysis based on a decade of data from a large medical platform, a random forest model demonstrated the best performance in predicting in-hospital mortality in elderly patients with HF and hypertension, achieving an AUC of 0.850, high recall (0.837), and a low Brier score (0.178). Shapley additive explanations (SHAP) analysis identified urea, length of stay, neutrophils, albumin, and HDL cholesterol as the most critical predictors (30).

In the present study, analysis of the NHANES database identified 1,598 individuals with HF, corresponding to a prevalence of 3.27%. According to the American Heart Association, the prevalence of HF among US adults is approximately 1.9% to 2.6% for the overall population and increases with advancing age (31). Compared with these official statistics, the prevalence observed in our study is modestly higher.Similarly, the clinical data set of 584 participants,132 patients were diagnosed with HF, corresponding to a prevalence of 22.6%. In both samples, patients with HF were older and had higher HbA1c/HDL-C ratios. They also exhibited a greater prevalence of hypertension, HF, and stroke compared to those without HF. Subgroup analyses indicated that the association between HF and these factors was particularly pronounced in participants aged ≥60 years (OR = 1.15, 95%CI: 1.05–1.25), with no significant interactions observed across subgroups. However, the association varied by diabetes status, and was more pronounced in participants with diabetes. Insulin resistance and compensatory hyperinsulinemia may represent key mechanistic links underlying the observed association between the HbA1c/HDL-C ratio and HF. Insulin resistance is characterized by impaired glucose utilization and is often accompanied by chronic low-grade inflammation, oxidative stress, and endothelial dysfunction, all of which contribute to adverse cardiac remodeling (32, 33). Meanwhile, hyperinsulinemia may promote sympathetic nervous system activation, sodium retention, and myocardial hypertrophy, thereby increasing the risk of HF.Furthermore, dysregulated lipid metabolism, reflected by low HDL-C levels, may exacerbate insulin resistance and impair reverse cholesterol transport, leading to lipotoxicity in cardiomyocytes (34). Therefore, the HbA1c/HDL-C ratio may capture the combined effects of impaired glycemic control, lipid dysregulation, and insulin resistance, providing a more integrated assessment of cardiometabolic risk.The HbA1c/HDL-C ratio represents a powerful composite marker, as it captures two critical and interacting pathways in HF pathophysiology: glucotoxicity and impaired cholesterol efflux (35). Chronic hyperglycemia, reflected by elevated HbA1c levels, promotes the formation of advanced glycation end products, which contribute to myocardial fibrosis and diastolic dysfunction (36). Simultaneously, low HDL-C levels (the denominator) signify reduced reverse cholesterol transport and diminished anti-inflammatory capacity, allowing for lipid accumulation and sustained inflammation in the myocardium (37). The synergistic effect of these two conditions, captured by their ratio, may create a “perfect storm” for cardiac damage, thereby accounting for the strong association observed (12).

Our study demonstrates that the nonlinear saturation pattern is biologically plausible and consistent across two different populations. Below the inflection point of 6.21, the myocardium appears highly sensitive to incremental increases in HbA1c/HDL-C ratio, suggesting a dose-dependent response to combined gluco-lipotoxicity. Once this critical threshold is exceeded,however, the underlying pathological processes may become fully activated and approach a state of saturation. Emerging evidence further indicates that elevated HbA1c/HDL-C ratios are significantly associated with an increased risk of cardiovascular and metabolic diseases in older adults.In a prospective cohort study of 7,256 middle-aged and older adults from CHARLS, Dong et al. (38) investigated the HbA1c/HDL-C ratio and depression with incident cardiometabolic multimorbidity (CMM).Higher HbA1c/HDL-C ratio and depression were each independently associated with an increased CMM risk (HR = 2.59, 95% CI: 1.82–3.68).In addition, a seven-year study of 5,165 participants using CHARLS data found that a higher cumulative mean HbA1c/HDL-C ratio was significantly associated with an increased risk of new-onset stroke (39).

Our study offers several noteworthy advantages. First, our findings were validated across two independent samples, enhancing their reliability and generalizability. Second, we elucidated a significant positive correlation between the HbA1c/HDL-C ratio and HF. Third, stratified and interaction analyses were performed to improve the stability of our findings.

However, several limitations of our research should also be considered. Firstly, a significant limitation stems from the cross-sectional design of our data, which inherently restricts our ability to establish a definitive causal relationship between the HbA1c/HDL-C ratio and HF. Secondly, the reliance on self-reported diagnoses of HF within the NHANES sample is a potential source of misclassification bias, as participant recall or understanding may not perfectly align with clinical diagnoses. Thirdly, while extensive adjustments for various covariates were performed, the possibility of residual confounding by unmeasured or imperfectly measured factors cannot be entirely ruled out. Finally, the inpatient sample was drawn from a single center, which may limit the generalizability of these findings to broader and more diverse patient populations. Future prospective cohort studies are required to shed more light on our results.

## Conclusions

In summary, the present research indicated that a significant and robust non-linear positive association between the HbA1c/HDL-C ratio and the risk of HF was observed, consistently across two distinct adult populations. These findings implicated that the HbA1c/HDL-C ratio could be a promising and effective biomarker for identifying individuals at risk of HF. Further large-scale research is urgent to demonstrate these associations and biological mechanisms.

## Data Availability

The original contributions presented in the study are included in the article/Supplementary Material, further inquiries can be directed to the corresponding authors.
