# Supplementary material for: Nonlinear association of HbA1c/HDL-C ratio with heart failure in adults: a large cross-sectional study
Source: Front Cardiovasc Med. 2026 Jun 3;13:1844807. doi: 10.3389/fcvm.2026.1844807 (PMC13271938; doi:10.3389/fcvm.2026.1844807)
Supplement: Supplementary file 1 [file Datasheet1.docx]

**Supplementary**

**Table S1 Baseline characteristics of included and excluded NHANES participants，weight**

| **Characteristic** | **Excluded participants** | **Included participants** | ***P*-value** |
| --- | --- | --- | --- |
| n | 52394 | 48922 |  |
| **Age (years)** | 14.54 ± 16.71 | 49.60 ± 18.16 | 0.017 |
| **Neutrophil, 10^9^ /L** | 3.70 ± 1.72 | 4.32 ± 1.79 | 0.007 |
| **TC, mg/dL** | 163.99 ± 31.10 | 203.66 ± 43.32 | 0.002 |
| **Gender,n(%)** |  |  | 0.259 |
| Male | 26309 (50.21) | 23584 (48.21) |  |
| Female | 26085 (49.79) | 25338 (51.79) |  |
| **Marital status,n(%)** |  |  | <0.001 |
| Married/Living with Partner | 3387 (6.46) | 29377 (60.05) |  |
| Widowed/Divorced/Separated | 1780 (3.40) | 10698 (21.87) |  |
| Never married | 7898 (15.07) | 8374 (17.12) |  |
| Missing | 39329 (75.06) | 473 (0.97) |  |
| **PIR,n(%)** |  |  | <0.545 |
| Poor | 15749 (30.06) | 9089 (18.58) |  |
| Nearly poor | 12905 (24.63) | 11961 (24.45) |  |
| Middle income | 11043 (21.08) | 12008 (24.55) |  |
| High income | 7743 (14.78) | 11622 (23.76) |  |
| Missing | 4954 (9.46) | 4242 (8.67) |  |
| **Education level,n(%)** |  |  | 0.754 |
| Below high school | 20832 (39.76) | 5897 (12.05) |  |
| High school | 11065 (21.12) | 18554 (37.93) |  |
| Above high school | 3670 (7.00) | 24407 (49.89) |  |
| Missing | 16827 (32.12) | 64 (0.13) |  |
| **Ethnicity,n(%)** |  |  | 0.084 |
| Non-Hispanic White | 15584 (29.74) | 21848 (44.66) |  |
| Non-Hispanic Black | 13778 (26.30) | 9866 (20.17) |  |
| Mexican American | 13767 (26.28) | 8682 (17.75) |  |
| Other Race | 9265 (17.68) | 8526 (17.43) |  |
| **Smoking behavior,n(%)** |  |  | <0.001 |
| Never | 4226 (8.07) | 26555 (54.28) |  |
| Former | 1465 (2.80) | 12151 (24.84) |  |
| Now | 1347 (2.57) | 10174 (20.80) |  |
| Missing | 45356 (86.57) | 42 (0.09) |  |
| **Drinking behavior,n(%)** |  |  | 0.259 |
| Never | 912 (1.74) | 6437 (13.16) |  |
| Former | 554 (1.06) | 7807 (15.96) |  |
| Now | 2108 (4.02) | 29804 (60.92) |  |
| Missing | 48820 (93.18) | 4874 (9.96) |  |
| **Hypertension,n(%)** | 2963 (9.81) | 20416 (41.74) | 0.130 |
| **CHD,n(%)** | 336 (5.56) | 2015 (4.13) | 0.227 |
| **Stroke,n(%)** | 381 (6.22) | 1884 (3.85) | 0.090 |
| **DM,n(%)** |  |  | 0.084 |
| Yes | 1108 (2.41) | 8459 (17.78) |  |
| Borderline | 320 (0.69) | 3452 (7.26) |  |

For continuous variables: survey-weighted mean (95% CI) , *P*-value was by survey-weighted linear regression
For categorical variables: survey-weighted percentage (95% CI) , *P*-value was by survey-weighted Chi-square test
Abbreviations:Q,Quartile; TC, Total Cholesterol (mg/dL);DM,Diabetes Mellitus;CHD,Coronary Heart Disease;

**TABLE S2 Association of HbA1c/HDL‑C ratio with HF in non‑DM and DM subgroups (NHANES).Adjusted for the variables listed in model 3 except for DM**

| **Subgroups** | Non-DM | | DM | |
| --- | --- | --- | --- | --- |
|  | OR(95CI) | *P* value | OR(95CI) | *P* value |
| HbA1c/HDL-C ratio | 1.12 (1.06, 1.19) | 0.001 | 1.10 (1.06, 1.13) | <0.001 |
| HbA1c/HDL-C ratio(quartiles) |  |  |  |  |
| Q1 | Reference |  | Reference |  |
| Q2 | 1.09 (0.86, 1.40) | 0.473 | 1.25 (0.88, 1.78) | 0.216 |
| Q3 | 1.22 (0.95, 1.55) | 0.114 | 1.24 (0.89, 1.73) | 0.205 |
| Q4 | 1.63 (1.27, 2.11) | 0.001 | 1.89 (1.39, 2.58) | <0.001 |
| *P* for trend |  | 0.001 |  | <0.001 |

Abbreviations:Q,Quartile; TC, Total Cholesterol (mg/dL);DM,Diabetes Mellitus;CHD,Coronary Heart Disease;OR: odds ratio, CI: confidence interval.

Model 3: Adjusted for age, gender, ethnicity, marital status, poverty income ratio, education level,smoking behavior, drinking behavior, hypertension, Stroke, neutrophils, TC, CHD.

**TABLE S3 Multivariate logistic regression between HbA1c/HDL-C ratio and HF using multiply imputed data (NHANES)**

| **Subgroups** | **Model1** |  | **Model2** |  | **Model3** |  |
| --- | --- | --- | --- | --- | --- | --- |
|  | OR(95CI) | *P* value | OR(95CI) | *P* value | OR(95CI) | *P* value |
| HbA1c/HDL-C ratio  (continuous) | 1.17 (1.16, 1.19) | <0.001 | 1.16 (1.14, 1.18) | <0.001 | 1.07 (1.05, 1.10) | <0.001 |
| HbA1c/HDL-C ratio  (quartiles) |  |  |  |  |  |  |
| Q1 | Reference |  | Reference |  | Reference |  |
| Q2 | 1.19 (1.08, 1.31) | 0.001 | 1.18 (1.07, 1.30) | 0.001 | 1.05 (0.95, 1.17) | 0.307 |
| Q3 | 1.44 (1.31, 1.58) | <0.001 | 1.41 (1.29, 1.55) | <0.001 | 1.12 (1.01, 1.24) | 0.029 |
| Q4 | 2.17 (1.99, 2.36) | <0.001 | 2.08 (1.90, 2.27) | <0.001 | 1.36 (1.23, 1.51) | <0.001 |
| *P* for trend |  | 0.001 |  | 0.001 |  | 0.001 |

Abbreviations:Q,Quartile; TC, Total Cholesterol (mg/dL);DM,Diabetes Mellitus;CHD,Coronary Heart Disease;OR: odds ratio, CI: confidence interval.

Model 1: Non-adjusted model.

Model 2: Adjusted for age, gender.

Model 3: Adjusted for age, gender, ethnicity, marital status, poverty income ratio, education level,smoking behavior, drinking behavior, hypertension, DM, Stroke, neutrophils, TC, CHD.

**
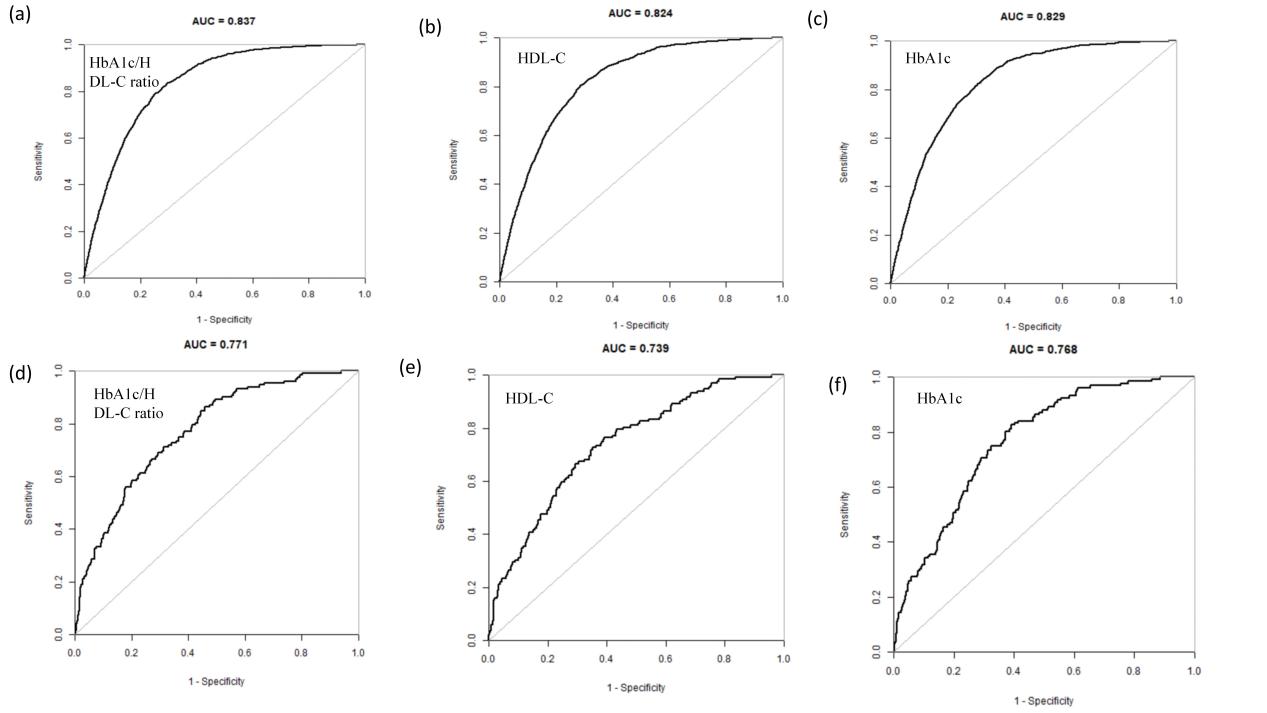
Figure S1 Comparison of receiver operating characteristic (ROC) curves for heart failure (HF) prediction.Panel (a)-(c) show ROC curves derived from the NHANES data, while panel (d)-(f) represent ROC curves from the clinic data.**

**Abbreviations: AUC, area under the curve. HbA1c,Glycated hemoglobin. HDL-C ,High-density lipoprotein cholesterol**
